# Trajectories of informal care and health

**DOI:** 10.1016/j.ssmph.2016.05.009

**Published:** 2016-07-19

**Authors:** A. Vlachantoni, J. Robards, J. Falkingham, M. Evandrou

**Affiliations:** a EPSRC Care Life Cycle, Social Sciences, University of Southampton, SO17 1BJ, UK; b ESRC Centre for Population Change, Social Sciences, University of Southampton, SO17 1BJ, UK; c Centre for Research on Ageing, Social Sciences, University of Southampton, SO17 1BJ, UK

**Keywords:** Ageing, Health status, Informal caring, Social care, Living arrangements, Census

## Abstract

The evidence of the impact of informal care provision on the health of carers presents a complex and contested picture, depending on the characteristics of the care studied, including its duration, which has been relatively short in previous research (up to 4 years). Drawing on data from the Office for National Statistics Longitudinal Study, a 1% sample of linked Census records for respondents in England and Wales (*N*=270,054), this paper contributes original insights on the impact of care provision on the carer's health ten years later. The paper explores differentials in self-reported health in 2011 between individuals according to their caring status at 2001 and 2011, and controlling for a range of demographic and socio-economic characteristics. The results show that individuals providing informal care in 2011 (regardless of carer status in 2001) exhibit lower odds of poor health in 2011 than those who did not provide care in both 2001 and 2011. Taking the intensity of care into account, ‘heavy’ carers in 2001 (i.e. caring for more than 20 h per week) who were not caring in 2011 show a higher likelihood of reporting poor health than non-carers, while those who were ‘heavy’ carers in both 2001 and 2011 are around one-third less likely to report poor health at 2011 compared to non-carers (2001 and 2011). These findings provide new insights in relation to repeat caring and its association with the carer's health status, further contributing to our understanding of the complex relationship between informal care provision and the carer's health.

## Highlights

• Carer and non-carer health outcomes are compared over a 10 year period.
• Starting/continuing light care is linked to a lower risk of poor health than not caring.
• Light carers who stopped caring showed no difference in the odds of poor health.
• Heavy carers who stopped caring were 22% more likely to report poor health.
• The timing and intensity of caring are directly associated with the carer's health.

## 1 Introduction

The provision of unpaid or informal care is an increasingly common experience, particularly at older ages, and an important component of social care in England and Wales (Doran et al., 2003, Pickard et al., 2000, Pickard, 2015, Hirst, 2002; Vlachantoni, 2010). Driven by increasing longevity and changes in living arrangements (Robards et al., 2012, Grundy and Tomassini, 2010, Norman and Purdam, 2013), informal caring in England and Wales increased at a faster pace than population growth between 2001 and 2011; the largest growth was among those providing 50 h or more care per week (Office for National Statistics, 2013a). From a social policy perspective, understanding health patterns among the carers' population is important as carers' health status is crucial both in relation to their ability to provide support and in terms of their own care need. Existing empirical evidence regarding the relationship between informal caring and health outcomes is mixed, depending on a range of factors such as the study type (cross-sectional vs. longitudinal) (Vlachantoni, Evandrou, Falkingham & Robards, 2013), the specific health outcome measured (Brown and Brown, 2014, Jones and Peters, 1992) and health characteristics of the person cared for (Capistrant, Moon, Berkman, & Glymour, 2012). Much of the research investigating care trajectories and their impact on the carer's physical or mental health has focused on relatively short time periods (see for example Burton, Zdaniuk, Schulz, Jackson, & Hirsch, 2003); less is known about the impact of caring for individuals at more than one time point in the life course, and where the time points are one decade apart. This study contributes to that part of the literature which aims to understand health outcomes among informal carers across different time points and compared to individuals who have not provided any informal care. Using the Office for National Statistics (ONS) Longitudinal Study (LS), a nationally representative 1% sample of linked Census data for England and Wales, this paper follows informal carers between 2001 and 2011 in order to explore their health status in 2011. By studying this relationship across 10 years, the paper contributes original insights into our understanding of the impact of different care trajectories on the health of the carer.

## 2 Previous research on informal care provision and health

Previous studies exploring the link between provision of informal care and health reflect the complexity of researching this topic area. Cross-sectional analyses may be limited in examining the factors preceding or following an individual's provision of informal care provision; however they can highlight the importance of distinguishing between particular types of care, or the importance of exploring the intensity of care provision (Evandrou, 1996). For example, analysis of 2001 UK Census data showed that non-carers were slightly more likely than carers to report good health (Doran et al., 2003). However, O’Reilly, Connolly, Rosato, and Patterson (2008), using data from the 2001 Northern Ireland Census, found that although carers were less likely than non-carers to report a limiting long-term illness (LLTI), health outcomes were worse among men providing 50 h of care per week or more. Similarly, Young, Grundy, and Kalogirou (2005), using 2001 census data for England & Wales and focussing on couples aged 65 and over in 2001 where at least one of the two spouses reported a LLTI, found that those who provided 20 h of care per week or more reported poorer health than those who provided fewer hours of care per week. More recent results analysing data from the 2011 UK Census indicate that informal carers are generally more likely to report ‘not good’ general health but that such likelihood increases in line with the hours of unpaid care provision, although this work does not control for the demographic characteristics of carers (Office for National Statistics, 2013, Office for National Statistics, 2013).

Longitudinal analyses can identify the effect of informal care provision on the carer's health, as well as their mortality risk (Vlachantoni, Evandrou, Falkingham & Robards, 2013). Studying informal carers at more than one point in time, identifying ‘care trajectories’, and their impact on individuals’ wellbeing is increasingly important in the context of both population ageing and increasing diversity in household structures (Robards, Evandrou, Falkingham and Vlachantoni, 2012), the combination of which can require individuals to manage or combine multiple economic and caring roles (Evandrou & Glaser, 2004). For instance, Rahrig Jenkins, Kabeto, and Langa (2009) explored the impact of informal care provision by spouses in 2000 on the carers’ health status two years later, and did not find a negative effect. In contrast, Lawton et al. (2000) studied over 600 women aged 65 and over for 4 years, and found that women who had cared for at least 12 months were more likely to report poor physical and mental health compared to those who had not provided any care or care of a shorter duration during that time. In relation to the health and mortality of carers, O’Reilly et al. (2008) used data from the 2001 Northern Ireland Census on the health status of informal carers in order to explore their mortality risk 4 years later, and found that, controlling for a range of demographic and socio-economic characteristics, caregivers had a lower risk than non-caregivers, however such risk increased among caregivers as the number of hours of care provided increased. A similar study for England and Wales, using a comparable dataset, found that carers were more likely to report poorer health at baseline, yet survival analyses showed that they were at a significantly lower risk of dying (Ramsay, Grundy, & O'Reilly, 2013). Indeed, similar research in the US found lower mortality among caregivers leading to the suggestion that it may be premature to conclude that health risks for caregivers are due to providing active help (Brown et al., 2009) and caregivers may benefit from providing care. Indeed some empirical work has drawn attention to the potential health benefits arising from informal carer roles, which may include improved self-worth, ‘proximity’ to a spouse and health benefits from ‘helping behaviour’ (Poulin et al., 2013, Kramer, 1997).

Longitudinal analyses have also stressed the importance of taking into account the specific characteristics of the caring activity, for example the type of care provided (e.g. personal and/or instrumental) and the health characteristics of the person being cared for. Research on the provision of informal care in England and Wales between 2001 and 2011 found that over one-third of carers in 2001 were also providing care after 10 years (Robards, Vlachantoni, Evandrou, and Falkingham, 2015), suggesting a high propensity to continue to provide care once such a role has been initiated. Research from the United States using the US Changing Lives of Older Couples survey has highlighted the importance of the duration of spousal care provision on the carer's psychological wellbeing following widowhood and found that care provision of a longer duration appears to have a more positive effect than shorter care provision (Keene & Prokos, 2008). Other research using the same dataset found that individuals who had provided instrumental support to friends, relatives and neighbours were 50% less likely to die in the following five years than those who had not provided any support (Brown, Nesse, Vinokur, & Smith, 2003). Interestingly, however, although much of the research highlights positive physical health outcomes, worse mental health outcomes have been noted as a result of caregiving responsibilities for heavy informal carers (providing 20 h or more of care per week) (Colombo, Llena-Nozal, Mercier, & Tjadens, 2011). Simon, Kumar, and Kendrick (2009) in a cohort study of 105 informal live-in carers of new stroke patients, found that informal carers were 2.5 times more likely to experience psychological distress than non-carers.

Taylor, Ford, and Dunbar (1995) critically evaluated a range of studies examining effects of caring on health and argued that selection into caring roles is an important consideration. An alternative, albeit smaller, body of research has therefore focused on the opposite direction of the relationship between care provision and health, exploring the effect of one's health status on their caring activity at a later point. For example, Young and Grundy (2008) analysed data from two Census points and found that individuals reporting a LLTI in 1991 and/or in 2001 were more likely to be providing informal care in 2001 than those not reporting a LLTI (Young & Grundy, 2008). Relating to these findings are those of Burton et al. (2003), who showed that, among 428 individuals studied at baseline and five years later, those with higher levels of health-risk behaviour were more likely to take up caring roles than those who did not report any such behaviour. By contrast, McCann, Grundy, and O'Reilly (2012) followed individuals for three years; respondents with good physical health were more likely to become caregivers and to continue caring, although such continuation of the caring role was also associated with declining mental health.

In summary, varying results on the relationship between caring and poor health have been identified from study to study leading to an ongoing debate on the relationship and the relative weight of ‘selection’ of individuals with worse health into the caring role because their worse health status makes them available to provide care to others (Brown & Brown, 2014). Against this background, the present paper aims to improve our understanding of the relationship between care trajectories and the carer's health, when such care is provided at more than one time points, and over the space of one decade. It investigates the association between past caring (2001) and present caring (2011), with poor health at 2011, after controlling for baseline health at 2001 and a range of demographic, socio-economic and area-based variables known to be associated with health. The key research question addressed in this paper is ‘how does the provision of informal care in 2001 and 2011, and the intensity of such care provision, affect the carer's report of poor health in 2011?’.

## 3 Data and methodology

The study uses data from the Office for National Statistics (ONS) Longitudinal Study (LS), a 1% extract of 2011 Census records matched to responses from the same individuals at the 2001 and earlier censuses. At the 2001 and 2011 Censuses questions were asked on self-reported health and on the provision of informal care. This paper thus examines poor health in 2011 with reference to the respondents’ current (2011) and previous (2001) provision of unpaid care, controlling for health at an earlier time point (2001) which may be related to an individual's selection into the original caring role or to one's health status earlier in a caring role (Brown and Brown, 2014). The question on the provision of informal care asked ‘*Do you look after, or give any help or support to family members, friends, neighbours or others because of either: long-term physical or mental ill-health/disability/ problems related to old age?*’ and requested respondents not to include any provision which was part of paid employment and to exclude childcare.

A binary logistic regression model was constructed with ‘poor health’ (recorded as bad or very bad health) at the 2011 Census as the outcome measure. The census question asked ‘*How is your health in general?*’ with five response options (very good, good, fair, bad, very bad). The analyses are restricted to a sample of those aged 25–74 years in 2001 and present also in 2011 (*N*=270,054); which constitutes the key informal caring age group (Office for National Statistics, 2013, Office for National Statistics, 2013) and avoids issues arising from 2001 post-census editing (Buxton and Smith, 2010). The model in this paper takes into account self-reported health and reporting a limiting long term illness (LLTI), and a range of factors which have been shown to be important in previous literature examining the relationship between the provision of informal care and the carer's health. These include demographic characteristics sex (Seale, 2000), age (Murphy, Grundy, & Kalogirou, 2007), ethnic group (Office for National Statistics, 2013, Office for National Statistics, 2013), marital status (Keene & Prokos, 2008); co-morbidities within the household (reporting of a LLTI within the household) (Burton et al., 2003); and socio-economic characteristics including housing tenure (McCann et al., 2012), area effects (Carstairs index) (Dorling and Thomas, 2009, Hanratty et al., 2007, Thomas et al., 2010, Sloggett and Joshi, 1994), highest educational qualification and car access. We also tried the inclusion in the model of social class and an adjusted economic activity variable, omitting the category of permanently sick individuals (*N*=11,706) which is directly related to the outcome variable, however these did not affect the direction and strength of the results, and therefore these variables were excluded from the analysis so as to maintain the largest possible analytical sample.

Due to the link between bereavement and poor health (Vlachantoni, Evandrou, Falkingham, Robards, 2013), the models also control for change in marital status between 2001 and 2011. The inclusion of information on whether there is another person with a limiting long term illness within the household (at 2001 or 2011) controls for co-morbidity, and may also act as an indicator of whether care is being provided within the same household (Norman & Purdam, 2013). All controls are measured in 2011, except for the respondents’ health and reporting a LLTI at baseline (2001), while the reporting of a LLTI by someone else in the household was measured at both 2001 and 2011, and changes in one's marital status were recorded between 2001 and 2011, in order to control for the effect of such transitions on the respondents’ health. The models were repeated for males and females enabling the identification of gender-specific associations between caring and health which have been noted in the past (Dahlberg, Demack, & Bambra, 2007). The analyses were completed in STATA 11.

## 4 Understanding patterns of informal care provision between 2001 and 2011

In order to understand patterns of informal care provision between 2001 and 2011, a typology of caring transitions was used to capture change (or the lack of it) in the provision of informal care between 2001 and 2011 and the transition between caring and non-caring roles over the decade (Groups a–d in Table 1). Building on existing research in order to highlight the importance of the intensity of care provision (Robards, Vlachantoni, Evandrou, Falkingham, 2015), the table also shows transitions between non-caring roles, light caring roles (defined as providing 1–19 h of care per week), and heavy caring roles (defined as providing 20 h or more informal care per week) (Groups 1–9 in Table 1).

**Table 1:** Typology of caring transitions between 2001 and 2011 Census.

**Group/description**	**2001 Census**	**2011 Census**	
**(a) Not caring at 2001 and 2011**			
1. *Non-carers*	Not caring		Not caring
**(b) Not caring at 2001, caring at 2011**			
2. *Non-carer to light carer*	Not caring		1–19 h/week
3. *Non-carer to heavy carer*	Not caring		20 h+/week
**(c) Caring at 2001, not caring at 2011**			
4. *Light carer to non-carer*	1–19 h/week		Not caring
5. *Heavy carer to non-carer*	20 h+/week		Not caring
**(d) Caring at 2001 and 2011**			
6.*Persistent light carer*	1–19 h/week		1–19 h/week
7. *Carer, increasing intensity*	1–19 h/week		20 h+/week
8. *Carer, decreasing intensity*	20 h+/week		1–19 h/week
9. *Persistent heavy carer*	20 h+/week		20 h+/week

Table 2 shows the distribution of the sample across the caring typologies. Of the total sample, 73.6% were not caring at both 2001 and 2011 (group a), with 11.4% transitioning into a caring role at 2011 from a non-caring role at 2001 (group b), while 9.6% were caring in 2001 and not caring ten years later (group c), and 5.4% were caring at both 2001 and 2011 (group d). Taking into account the number of hours of care provided, 1.5% of the sample were ‘persistent heavy carers’, providing 20 h or more of care in both 2001 and 2011. The final column of Table 2 presents the proportion within each carer group reporting poor health in 2011, which merges the categories of bad and very bad health. Overall, 8.3% of the sample reported poor health in 2011. Interestingly however, there are significant differences in the prevalence of poor health across the different caring typologies, with the highest prevalence found amongst those who moved from providing heavy care in 2001 to no care in 2011 (group 5) (17.1%). The direction of causality, i.e. whether poor health led to the cessation of caring or vice versa, is difficult to ascertain. It is, however, apparent from Table 2 that the prevalence of poor health is also higher than the population average amongst those who had moved from no caring in 2001 to heavy caring in 2011 (group 3) (12.6%) and among persistent heavy carers (group 9) (14.3%). It is important to note that these results are obtained before any standardisation for health at baseline or the age of the ONS LS member, highlighting the need for multivariate analyses.

**Table 2:** Typology of caring amongst ONS LS members aged 35–85 years (2011) resident at both 2001 and 2011 Censuses; and percentage of each care group reporting poor health.

Carers typology	%	*N*	% of care group reporting poor health in 2011
**(a) Not caring at 2001 and 2011**	**73.6**	**198,753**	**8.2**

**(b) Not caring at 2001, caring at 2011**	**11.4**	**30,682**	**11.4**
*2. Non-carer to light carer*	*7.6*	*20,559*	*4.3*
*3. Non-carer to heavy carer*	*3.7*	*10,123*	*12.6*

**(c) Caring at 2001, not caring at 2011**	**9.6**	**25,939**	**9.6**
*4. Light carer to non-carer*	*7.2*	*19,338*	*7.8*
*5. Heavy carer to non-carer*	*2.4*	*6601*	*17.1*

**(d) Caring at 2001 and 2011**	**5.4**	**14,680**	**5.4**
*6. Persistent light carer*	*2.5*	*6747*	3.5
*7. Carer, increasing intensity*	*0.9*	*2466*	9.5
*8. Carer, decreasing intensity*	*0.5*	*1305*	8.5
*9. Persistent heavy carer*	*1.5*	*4162*	14.3

**Total**	**100%**	**270,054**	**8.3%**

## 5 Exploring the association between caring, repeat caring and the carer's health status

Table 3 presents the odds ratios for reporting poor health at 2011 for the different caring typologies. Among those who were not caring at 2001 and caring at 2011 (group b), there are substantially lower odds of reporting poor health at 2011 (OR 0.64, 95% CI 0.60–0.67). Similarly, those caring at both 2001 and 2011 (group d) show lower odds of reporting poor health at 2011 (OR 0.56, 95% CI 0.52–0.60), suggesting that all informal carers at 2011 are less likely to report poor health than persistent non-carers, even after controlling for the full range of characteristics associated with health, socio-economic characteristics, changes in marital status, co-morbidities within the same household, area effects and baseline health. However, amongst those caring at 2001 and not caring at 2011 (group c), the odds of reporting poor health are around 10% higher (OR 1.09, 95% CI 1.04–1.15) than non-carers (reference category). This finding may point to a negative impact of caring on health which necessitates, or contributes to, the cessation of the caring role. The analysis was also run without the variable indicating co-morbidities within the same household, and the results were broadly similar except that the positive impact of caring on the health of those presumed to be caring for individuals *outside* their household, was even stronger.

**Table 3:** Regression odds ratios for reporting poor health at 2011 among ONS LS members aged 35–85 years (2011) resident at 2001 and 2011.

**Carer status**	**Model 1**				**Model 2**			
	**OR**	***P* (sig)**	**95% CI**		**OR**	***P* (sig)**	**95% CI**	
**(a) Not caring at 2001 and not caring at 2011**	1							
1. Non-carer					1			
**(b) Not caring at 2001, caring at 2011**	0.64	0.000	0.60	0.67				
2. Non-carer to light carer					0.58	0.000	0.54	0.63
3. Non-carer to heavy carer					0.69	0.000	0.64	0.74
**(c) Caring at 2001, not caring at 2011**	1.09	0.001	1.04	1.15				
4. Light carer to non-carer					1.03	0.390	0.97	1.09
5. Heavy carer to non-carer					1.22	0.000	1.13	1.33
**(d) Caring at 2001 and 2011**	0.56	0.000	0.52	0.60				
6. Persistent light carer					0.45	0.000	0.50	0.68
7. Carer, increasing intensity					0.58	0.000	0.50	0.68
8. Carer, decreasing intensity					0.64	0.000	0.51	0.80
9. Persistent heavy carer					0.63	0.000	0.56	0.70

Table 3 further decomposes these differentials, highlighting the importance of taking the intensity of care provision into account. In particular those who transitioned from a heavy carer's role in 2001 to being a non-carer in 2011 (group 5) had significantly higher odds of reporting poor health at 2011 (OR 1.22, 95% CI 1.13–1.33), whereas those moving from light care to non-care (group 4) did not have significantly different odds to the reference group (OR 1.03, 95% CI 0.97–1.09). In contrast, all those who provided care at both time points or took up a caring role in 2011 showed lower odds of reporting poor health, regardless of the intensity of such care provision.

Persistent light carers (group 6) exhibit the best health outcomes (OR 0.45, 95% CI 0.39–0.52). Among light carers at 2001 who were then providing heavy care at 2011 (group 7) and non-carers at 2001 who were light carers at 2011 (group 2), the odds of reporting poor health at 2011 are identical (OR 0.58, 95% CI 0.50–0.68 and OR 0.58, 95% CI 0.54–0.63 respectively). These two groups show lower odds of reporting poor health compared to those who were not caring at 2001 and 2011. Notable is that these two groups experience a progressive increase in caring intensity from 2001 to 2011. In this respect, it is interesting to compare these two groups to the slightly higher odds of reporting poor health among non-carers at 2001 who were providing 20 h or more care at 2011, (i.e. who moved from providing no care to providing heavy care (OR 0.69, 95% CI 0.64–0.74)). Overall, all three groups still show lower odds of reporting poor health at 2011 compared to those not providing care in both 2001 and 2011. The respondents’ selection into the caring role might be an important factor relating to their own health status and the ability to care; this has been controlled for among non-carers at 2001, although it is not possible to do the same among carers at 2001 as they were already providing care at that point (and there is no suitable control variable from the 1991 Census).

**Fig. 1:**
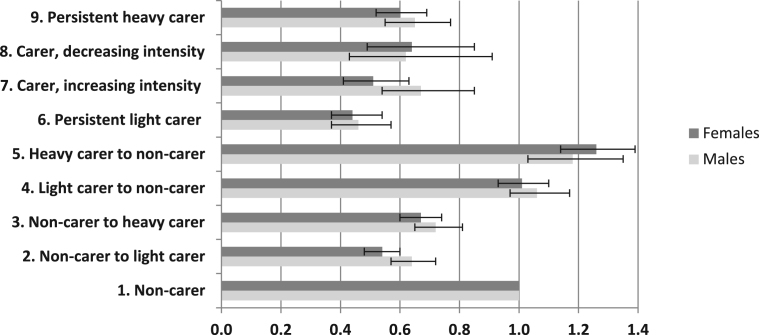
Regression odds ratios for reporting poor health at 2011 among ONS LS members aged 35–85 years (2011) resident at 2001 and 2011, by gender.

*Source*: Authors’ own analysis of ONS LS. Controls for health at baseline (2001 Census), limiting long term illness at baseline (2001), sex, age (2011), ethnic group (2011), change in marital status (2001–2011), housing tenure (2011), highest educational qualification (2011), car access (2011) and household limiting long term illness (2001 and 2011), Carstairs Index (2011).

The final model was re-run separately for men and women in order to identify gender-specific differences in the odds of reporting poor health. The results by gender presented in Fig. 1 show similar odds ratios across the carer groups, with some noteworthy exceptions. Gender differences are apparent for non-carers who transitioned to light caring (group 2), where men show higher odds (OR 0.64, 95% CI 0.57–0.72) than women (OR 0.54, 95% CI 0.48–0.60). There are also differences among those moving from light caring to heavy caring for men (OR 0.67, 95% CI 0.54–0.85) and women (OR 0.51, 95% CI 0.41–0.63).

## 6 Discussion and conclusion

Our understanding of the relationship between the provision of informal care and the carer's own health has often been constrained by small sample sizes or a relatively short period of study. The analysis presented in this paper has overcome these challenges by using the ONS LS dataset linking information between 2001 and 2011. The paper investigates the association between past and present informal care provision and poor health, controlling for the demographic, household and socio-economic characteristics of the respondents. The findings of this study are consistent with longitudinal studies from the US on health outcomes for informal carers, benefitting from a larger sample size and longer follow-up period than has been the case previously (Lawton et al., 2000; Rahrig Jenkins et al., 2009). The typology of care transitions highlights lower odds of poor health among informal carers at 2011 compared to non-carers at both 2001 and 2011. Among the key findings from these analyses is that the only two carer groups with higher odds of reporting poor health than non-carers (or with non-statistically significant results from the non-carers at 2001 and 2011) were those who transitioned from a caring to non-caring role between 2001 and 2011. All carers at 2011, regardless of the intensity of their care provision, showed lower odds of reporting poor health than non-carers at both 2001 and 2011. Notably, this includes those who were not caring in 2001 yet had begun caring by 2011 and for whom we have controlled for baseline health status in 2001 which could relate to selection of healthier individuals into a caring role. Light informal carers at 2001 who were no longer caring at 2011 showed no difference in the odds of reporting poor health compared to non-carers at either time point, suggesting no link between health outcomes and the cessation of the caring role. Such results are consistent with previous studies finding a positive health status for those who provide light care, a lower mortality risk amongst light informal carers (O’Reilly et al., 2008; Office for National Statistics, 2013, Office for National Statistics, 2013) and a lack of an improvement in health following the end of the caring role (Keene & Prokos, 2008). This may reflect the relatively less demanding nature of a less intense caring role, which may be directly related with both the carer's health status and the health status of the person cared for. In contrast, heavy carers at 2001 who were not caring at 2011 exhibited 22% higher odds of reporting poor health at 2011 than non-carers. It is likely that a large proportion of those providing heavy care in 2001 who stopped caring before 2011 did so because of the death of a (co-resident) spouse. The direction of causality in the relationship between stopping heavy caring and reporting poor health is difficult to identify conclusively, however the results are suggestive of a clear link between heavy caring and poor health outcomes, with modest differences between men and women.

The results from this study suggest that it is crucial to take into account the intensity of care provision when considering the health outcomes associated with a particular caring role. Questions on the provision of informal care and self-reported health were important additions to the 2001 and 2011 UK Censuses, and contribute to the empirical evidence base in this area. The ability to follow-up on individuals over a ten year period using data such as in the ONS LS highlights the importance of the UK longitudinal studies and the repetition of questions in the census from one time point to the next for issues of high public policy importance, such as informal care provision.

Although the findings in this paper contribute new insights to our understanding of the complex relationship between informal care provision and health, nevertheless the study poses certain limitations which necessitate caution in the interpretation of the findings. Firstly, the nature of the data which is drawn from the UK Census means that it is not possible to determine the duration of the caring role between the two time points. Therefore, it is possible that an individual who is providing care at both 2001 and 2011 may have experienced periods of non-caring over the decade between the two Censuses, just as an individual who is classified as a non-carer at both 2001 and 2011 may have provided care for a period of time between the two Census points. Such data limitations mean that we can only interpret the findings and examine the dynamics of informal care provision in relation to the two time points and not in the in-between period; thus caring at both 2001 and 2011 is best viewed as a repeated rather than a continuous activity. Secondly, the dataset does not allow for the identification of the care recipient or the exploration of the quality of the relationship between the carer and the care recipient, both of which are important dimensions affecting the caring activity (Keene & Prokos, 2008). Information on the nature of the care provided (e.g. physical, psychological, emotional) is also lacking, which may have a bearing on the association under study (Brown et al., 2003, Maher and Green, 2002). Thirdly, no information is known on the carer's access to formal support or their use of services provided by the state or purchased in the private sector, which may affect their provision of informal care in relation to specific tasks (e.g. employment of extra care assistance within the home) (Robards, Vlachantoni, Evandrou, and Falkingham, 2015). Such support may ‘buffer’ any adverse impacts of caring on the health of the carer. Fourthly, although the use of the self-reported measures of general health and the report of a LLTI have proven to be reliable indicators of individuals’ health status (Doran et al., 2003), nevertheless a more detailed examination of the carer's health would require additional measures of both their physical and mental condition (see for example Kenny, King, & Hall, 2014). Finally, the fact that we are able to investigate the relationship between caring and the carer's health over time does not allow us to draw conclusions about the direction of causality between the two, and due caution should be applied in the interpretation of results in Table 3 and Fig. 1 in this paper.

This study has highlighted that it is not only care provision per se which is associated with the carer's health status, rather the intensity and timing of such caring are also significant factors to take into account. As such, the study contributes to a better understanding of the nuanced and complex relationship between informal care provision and health, and provides a reminder that disentangling the effects of caring requires careful consideration of the context-specific characteristics of such relationship.

## Acknowledgements and funding

The authors wish to acknowledge the support of colleagues in the Engineering and Physical Sciences Research Council (EPSRC) Care Life Cycle (CLC) project (Grant number EP/H021698/1) and the Economic and Social Research Council (ESRC) Centre for Population Change (CPC) (Grant numbers RES-625-28-0001 and ES/K007394/1) at the University of Southampton.

The permission of the Office for National Statistics to use the Longitudinal Study is gratefully acknowledged, as is the help provided by staff of the Centre for Longitudinal Study Information & User Support (CeLSIUS). CeLSIUS is supported by the ESRC Census of Population Programme (Award Ref: ES/K000365/1). The authors alone are responsible for the interpretation of the data.

This work contains statistical data from ONS which is Crown Copyright. The use of the ONS statistical data in this work does not imply the endorsement of the ONS in relation to the interpretation or analysis of the statistical data. This work uses research datasets which may not exactly reproduce National Statistics aggregates.

We are grateful for the assistance of user support officers Julian Buxton, Lorraine Ireland, Shayla Leib, Kevin Lynch, Nicola Rogers, James Warren and the ONS LS Development Team for the extract from the dataset provided, their guidance on the dataset, and clearance of outputs.

The Carstairs Index has previously been used in conjunction with the ONS LS (see Boyle, Norman and Rees, 2004; Norman & Boyle, 2014; Norman, Boyle and Rees, 2005).
